# Influenza, PCV13, and PPSV23 Vaccination Rates Among Inflammatory Bowel Disease Patients With Additional Co-Morbidities as per CDC Recommendations

**DOI:** 10.7759/cureus.18387

**Published:** 2021-09-29

**Authors:** Ariel Jordan, Krystal Mills, Timothy Sobukonla, Alexander Kelly, Michael Flood

**Affiliations:** 1 Internal Medicine, University of Michigan, Ann Arbor, USA; 2 Internal Medicine, Morehouse School of Medicine, Atlanta, USA

**Keywords:** : inflammatory bowel disease, vaccines, influenza, pneumonia, african american

## Abstract

Background

Inflammatory bowel disease (IBD) and its immunosuppressive therapy alter the body's immune response, predisposing patients to higher infection risk preventable with vaccination. The CDC recommends every adult receive the annual influenza vaccine and patients with certain comorbidities receive the pneumococcal conjugate vaccine (PCV13) and pneumococcal polysaccharide vaccine (PPSV23). However, vaccination rates among IBD patients remain unacceptably low. The aim of our study is to present influenza and pneumococcal vaccinations rates of IBD patients at our center.

Methods

We hypothesized that vaccination rates will be suboptimal at our outpatient center and that patients are not being vaccinated based on comorbid conditions in accordance with guidelines. We retrieved electronic medical records from the gastroenterology clinic between December 2018 and December 2019. Data regarding influenza and pneumococcal vaccines, immunosuppressive drugs, and comorbidities were obtained. Microsoft Excel and SPSS Statistics (IBM Corp., Armonk, NY) were used for data analyses. A p-value < 0.05 was considered statistically significant.

Results

In total, 109 IBD patients were identified, 46.8% female and 53.2% male. The majority were African American (77.06%). The mean age was 45 years. Around 26.61% of the patients were on immunosuppressive therapy. Around 28.7% received the annual influenza vaccine, 42.2% PPSV23 alone, 19.27% PCV13 alone, and 16.5% received both. Patients >50 years were more likely to receive the influenza vaccine (P = 0.0122). Patients on immunosuppressive therapy were not more likely to be vaccinated with both PCV13 and PPSV23 (P = 0.1848, P = 0.7382). Active smokers were not more likely to be vaccinated with PPSV23 (P = 0.695). Patients with human immunodeficiency virus (HIV), chronic kidney disease (CKD), and sickle-cell disease were more likely to be vaccinated with both PCV13 and PPSV23 (P = 0.02, P = 0.02). Patients with other chronic medical conditions were more likely to be vaccinated with PPSV23 (P = 0.0201).

Conclusion

Our study revealed suboptimal influenza and pneumococcal vaccination rates among IBD patients at our facility. We also found that patients were not consistently vaccinated based on qualifying co-morbid conditions. Age plays a role in whether patients received the influenza vaccine contrary to guidelines. We urge clinicians to examine IBD patient vaccination rates at their facilities.

## Introduction

Vaccinations are a key element in supporting our immune system fight preventable infectious disease. The Centers for Disease Control and Prevention (CDC) provide a timetable regarding vaccination recommendations based on age and certain medical comorbidities that make one most susceptible to contracting that particular disease. Two conditions that are highly preventable for the full spectrum of age with appropriate vaccination are influenza and pneumococcal pneumonia. The CDC recommends that all patients above six months of age receive an annual influenza vaccine [1]. The CDC also recommends that all patients aged 65 or above receive both the pneumococcal conjugate vaccine (PCV13) and pneumococcal polysaccharide vaccine (PPSV23) in addition to patients with certain chronic medical conditions (i.e. diabetes, chronic heart, lung, or liver disease), immunocompromised health status, and/or certain social habits (i.e. cigarette smoking, alcoholism) [1].

Patients with certain chronic conditions are at increased risk of developing infectious diseases with higher rates of severe complications and mortality. By vaccination, these patients experience fewer hospitalizations and incur lower costs related to healthcare expenditure; this benefit of influenza and pneumococcal vaccines has been well studied. One systematic review demonstrated evidence that administration of the influenza vaccine to chronic obstructive pulmonary disease (COPD) patients was associated with a decrease in influenza related-COPD exacerbations and hospitalizations [2]. Another review of outcomes of influenza vaccination rates among patients with chronic health conditions found those vaccinated had a 62% reduction in outpatient sick visits and an 84% reduction in acute myocardial infarction hospitalizations [3]. A study investigating the efficacy of pneumococcal vaccine prophylaxis in COPD and congestive heart failure (CHF) patients found that vaccinated patients had a 74-84% reduction in healthcare expenses [4]. Additionally, patients with a compromised immune system due to an immunosuppressive disease (i.e. HIV) or therapy (i.e. TNF inhibitors [infliximab], anti-metabolites [methotrexate]) are at increased risk of developing more systemic and invasive versions of these typically mild diseases. One Canadian study found that immunocompromised persons were at 12x the risk and persons on immunosuppressive therapies were at 2.1-2.7x increased risks of developing invasive pneumococcal disease [5].

As previously stated, the data regarding influenza and pneumococcal vaccination rates among IBD patients have previously not been as well studied as for other chronic diseases. Therefore, the objectives of this study are 1) to examine influenza and pneumococcal vaccination rates among IBD patients at our outpatient center and 2) to identify areas of improvement and provide recommendations that can be utilized by clinicians at all centers servicing this patient population encouraging and ensuring vaccination uptake. We hypothesized that vaccination rates will be suboptimal at our outpatient center. Additionally, we hypothesized patients are not being vaccinated with PCV13 and/or PPSV23 based on additional comorbid medical conditions in accordance with CDC guidelines.

## Materials and methods

Participants

A total of 109 patients met our inclusion criteria. Inclusion criteria included patients aged 18 and above with a diagnosis of ulcerative colitis (UC) or Crohn’s Disease. Exclusion criteria were patients with indeterminate colitis and microscopic colitis. These patients were selected from the outpatient gastroenterology clinic of a large safety net hospital seen between December 2018 and December 2019. We retrieved and reviewed these patients’ electronic health records. Because of the observational nature of this study, the participants did not go through the informed consent process and were not compensated.

Vaccination status

The electronic health records of the included patients were reviewed to determine immunization/vaccination status. Specifically, influenza vaccine from the previous year, PCV13, and PPSV23 were documented. Additionally, information regarding immunosuppressive medications and medical comorbidities were captured and documented. This information was then compared to current CDC recommendations to determine appropriate immunization. 

Patient characteristics

Patient age, sex, race, insurance status (Medicare, Medicaid, private, or uninsured), IBD status (ulcerative colitis or Crohn’s Disease), medical comorbidities (asthma, coronary artery disease, chronic kidney disease, COPD, liver disease, sickle cell disease, diabetes mellitus, HIV, hypertension, sarcoidosis of lung, CHF, cerebral vascular accident, and atelectasis), smoking, and alcohol status were obtained from the electronic medical record. 

Statistical analysis

Univariate statistics were computed for all variables. Microsoft Excel was used to organize extracted patient information for the following variables: Age, sex, race, occupation, insurance status, IBD status, co-morbid medical conditions, PCV13 and PCV23 vaccination status, influenza vaccination status, and use of immunosuppressive medications (corticosteroids, i.e. budesonide; biologics, i.e. ustekinumab; biosimilars, i.e. infliximab), immunomodulators (i.e. azathioprine, methotrexate). SPSS Statistics (IBM Corp., Armonk, NY) was used to stratify analysis. Correlations between rates of vaccination and age, sex, IBD status, immunosuppressive therapy, smoking, alcoholism, and comorbid conditions were obtained. A p-value of < .05 was considered statistically significant.

## Results

Baseline characteristics are provided in Table 1. There was a relatively even distribution of female (46.8%) and male (53.2%) patients. The mean age was 45 years with a standard deviation of 13.4 years. The majority of the patients had Crohn’s Disease (72.5%) and were African American (77.06%). Almost half of the patients were uninsured (44.95%) and the majority were unemployed (72.48%). Over half of the patients were non-smokers (65.14%) and the majority reported no use of alcohol (95.41%). Around 26.61% of patients were on immunosuppressive medications. Additionally, 28.7% of patients had received the influenza vaccine within the most recent influenza season, 19.27% had received the PCV13 vaccine, and 42.2% had received the PPSV23 vaccine. Around 34.86% had at least one of the listed comorbidities, in addition to IBD. Individual values for each comorbidity are detailed in Table 1.

**Table 1:** Characteristics of patient demographics and vaccination status (N=109).

	Frequency	Percent
Age		
50-69	40	36.7
<=49	66	60.55
>=70	3	2.75
Mean (SD)	45 (13.4)	
Gender		
Female	51	46.8
Male	58	53.2
Race		
African American	84	77.06
Caucasian	13	11.93
Other	12	11.01
IBD Status		
Crohn’s	79	72.5%
Ulcerative colitis	30	27.5%
Employment		
Employed	30	27.52
Not employed	79	72.48
Insurance		
Medicaid	18	16.51
Medicare	22	20.18
None	49	44.95
Private	20	18.35
Smoker		
No	71	65.14
Yes	38	34.86
Alcoholism		
No	104	95.41
Yes	5	4.59
Immunosuppressive medications		
No	80	73.39
Yes	29	26.61
Influenza		
No	77	71.3
Yes	31	28.7
PCV13		
No	88	80.73
Yes	21	19.27
PPSV23		
No	63	57.8
Yes	46	42.2
Comorbidity		
No	71	65.14
Yes	38	34.86
Asthma	6	15.79
Coronary artery disease	3	7.89
Chronic kidney disease	4	10.53
Chronic obstructive pulmonary disease	2	5.26
Liver disease	1	2.63
Sickle cell disease	2	5.26
Sarcoidosis of lung	1	2.63
Diabetes mellitus	12	31.58
Hypertension	1	2.63
HIV	31	81.58
Congestive heart failure	1	2.63
Cerebral vascular accident	1	2.63
Atelectasis	1	2.63

Table 2 provides the significance of vaccination status in relation to age, sex, race, insurance status, employment status, smoking, alcohol use, immunosuppressive medication use, additional comorbidities, and IBD type. Patients above the age of 50 were more likely to be vaccinated with the influenza vaccine than those under the age of 49 (p=0.0122). Age did not significantly impact those vaccinated with the PCV13 vaccine (p=0.0811). Patients above the age of 50 were also more likely to be vaccinated with the PPSV23 vaccine than those under the age of 49 (p=0.0482). Gender did not significantly impact vaccination with the influenza vaccine (p=0.1946), the PCV13 vaccine (p=0.352), or the PPSV23 vaccine (p=0.2867). Race did not significantly impact vaccination rate for the influenza vaccine (p=0.9018), PCV13 vaccine (p=0.8918), or PPSV23 vaccine (p=0.3217). Employment status did not significantly impact patients’ influenza vaccine (p=0.8535) or PCV13 vaccine status (p=0.9047). However, unemployed patients were more likely to have received the PPSV23 vaccine than those employed (p=0.014). Insurance (type or lack thereof) did not significantly impact vaccination with the influenza (p=0.6116), PCV13 (p=0.5825), or PPSV23 vaccines (p=0.2359). Smoking was not associated with a greater likelihood of vaccination with the PPSV23 vaccine (p=0.0695). Immunosuppressive medications were not associated with a greater likelihood of vaccination with the PCV13 (p=0.1848) or PPSV23 vaccines (p=0.7382). Patients with additional comorbidities were more likely to be vaccinated with the PCV13 (p=0.0171) and PPSV23 vaccines (p=0.0201) but not the influenza vaccine (p=0.1634). Ulcerative colitis patients were more likely to receive the PCV13 vaccine (p=0.0045) while Crohn’s disease patients were more likely to receive the PPSV23 vaccine (p=0.0204).

**Table 2:** Statistical analysis

	Influenza			PCV13			PPSV23		
	No (N=77)	Yes (N=31)	p value	No (N=88)	Yes (N=21)	p value	No (N=63)	Yes (N=46)	p value
Age			0.0122			0.0811			0.0482
50-69	22(28.57)	17(54.84)		28(31.82)	12(57.14)		17(26.98)	23(50.00)	
<=49	53(68.83)	13(41.94)		57(64.77)	9(42.86)		44(69.84)	22(47.83)	
>=70	2(2.60)	1(3.23)		3(3.41)	0(0.00)		2(3.17)	1(2.17)	
Gender			0.1946			0.352			0.2867
Female	34(44.74)	16(59.26)		38(45.78)	12(57.14)		32(52.46)	18(41.86)	
Male	42(55.26)	11(40.74)		45(54.22)	9(42.86)		29(47.54)	25(58.14)	
Race			0.9018			0.8918			0.3217
African American	60(77.92)	24(77.42)		67(76.14)	17(80.95)		46(73.02)	38(82.61)	
Caucasian	9(11.69)	3(9.68)		11(12.50)	2(9.52)		10(15.87)	3(6.52)	
Other	8(10.39)	4(12.90)		10(11.36)	2(9.52)		7(11.11)	5(10.87)	
Employment			0.8535			0.9047			0.014
Employed	21(27.27)	9(29.03)		24(27.27)	6(28.57)		23(36.51)	7(15.22)	
Not employed	56(72.73)	22(70.97)		64(72.73)	15(71.43)		40(63.49)	39(84.78)	
Insurance			0.6116			0.5825			0.2359
Medicaid	12(15.58)	6(19.35)		16(18.18)	2(9.52)		8(12.70)	10(21.74)	
Medicare	17(22.08)	5(16.13)		16(18.18)	6(28.57)		10(15.87)	12(26.09)	
None	32(41.56)	16(51.61)		39(44.32)	10(47.62)		32(50.79)	17(36.96)	
Private	16(20.78)	4(12.90)		17(19.32)	3(14.29)		13(20.63)	7(15.22)	
Smoker			0.1953			0.87			0.695
No	47(61.04)	23(74.19)		57(64.77)	14(66.67)		42(66.67)	29(63.04)	
Yes	30(38.96)	8(25.81)		31(35.23)	7(33.33)		21(33.33)	17(36.96)	
Alcoholism			0.1132			0.2288			0.0798
No	75(97.40)	28(90.32)		85(96.59)	19(90.48)		62(98.41)	42(91.30)	
Yes	2(2.60)	3(9.68)		3(3.41)	2(9.52)		1(1.59)	4(8.70)	
Immunosuppressive Medications			0.8764			0.1848			0.7382
No	5672.73)	23(74.19)		67(76.14)	13(61.90)		47(74.60)	33(71.74)	
Yes	21(27.27)	8(25.81)		21(23.86)	8(38.10)		16(25.40)	13(28.26)	
Comorbidity			0.1634			0.0171			0.0201
No	53(68.83)	17(54.84)		62(70.45)	9(42.86)		51(80.95)	20(43.48)	
Yes	24(31.17)	14(45.16)		26(29.55)	12(57.14)		12(19.05)	26(56.52)	
IBD Status			0.2566						
Crohns	58(75.32)	20(64.52)		69(78.41)	10(47.62)		51(80.95)	28(60.87)	0.0204
UC	19(24.68)	11(35.48)		19(21.59)	11(52.38)	0.0045	12(19.05)	18(39.13)	

## Discussion

This retrospective single-center study revealed that overall influenza, pneumococcal, and thereby vaccination uptake in patients with IBD were low, which supported our hypothesis. This is of concern to the authors as, similar to other autoimmune diseases, which lead to dysregulated immunity, IBD patients are thought to be at increased risk of developing more invasive forms of vaccine-preventable diseases (VPD), such as influenza and pneumococcal pneumonia. Treatment regimens for IBD may include immunosuppressive therapies, such as TNF inhibitors and corticosteroids, which further contribute to altered immune systems that heighten the risk of these infections.

While there are no vaccination guidelines specific to IBD patients, a systematic review compiled the recommendations of various sources and came to a consensus regarding vaccination recommendations for IBD patients. This states that all IBD patients should receive an annual influenza vaccine, typically the inactivated form [6]. It has been shown that the influenza vaccine yields high seroprotection rates in adult patients with IBD. However, it is important to note that this seroprotection was decreased if the adult patients were receiving TNF inhibitors for IBD. For this reason, a high dosage of the influenza vaccine has been recommended for this cohort [7-9]. 

The aforementioned systematic review also revealed that if the patient received four doses of the pneumococcal vaccine in childhood, the first booster dose should occur five years after the last dose was administered as well as lifetime revaccination at the age of 65. If the adult never received any pneumococcal vaccinations, two doses should be given eight weeks apart, firstly PCV13 followed by PPSV23 [6]. Patients with IBD have been found to be at a higher risk for pneumonia, independent of immunosuppressive therapy [10]. It has been shown that though the pneumococcal vaccine is beneficial among patients with IBD, like the influenza vaccine, there is some reduced immunogenicity in IBD patients on immunosuppressive therapy [11].

Overcoming barriers to influenza and pneumococcal vaccination in patients with IBD and promoting vaccination uptake will improve the overall immunization rates in this cohort. At a provider level, it has been previously theorized that this may be a result of IBD patients not receiving preventative healthcare services at the same rate as other patients [12]. They found that only 71% of IBD patients in their study received routine primary care services in comparison to 78% of their general medicine counterparts. Another proposed theory is that gastroenterologists usually assume that these vaccinations are given by primary care providers and most GI clinics do not have the infrastructure to save vaccines [13]. They found that only 14.3% of gastroenterologists in their study admit to obtaining an immunization history from their patients and only one out of 164 patients were asked for a detailed immunization history, including vaccines from childhood. Another contributing factor may be that the CDC does not provide specific recommendations for IBD patients regarding a vaccination timetable. Thus, physicians are left to ponder how best to vaccinate IBD patients, specifically those on immunosuppressive regimens. 

At a patient level, it has been shown that the overall seasonal influenza vaccination rates for the US population remain below 70% [14]. One study of the general population identified factors such as older age, non-Hispanic white race and higher income with access to health insurance coverage have been linked to higher seasonal influenza vaccination rates [15]. However, other studies have shown that even controlling for variables such as increased risk, age distribution, perceived health status, poverty level, education, and access to medical care and health insurance, African Americans still receive disproportionately fewer vaccinations [16,17]. Focus group interviews have revealed that patients felt they were not routinely informed about or recommended by their physicians to receive influenza and pneumococcal vaccinations [18]. Of note, this study was conducted for an outpatient clinic at a safety-net US hospital, with non-Hispanic whites representing 11.93% of the cohort. Other races therefore comprised the majority, with African Americans accounting for 77.06%. Further research should be done to identify any patient-reported factors, which may account for the low uptake of vaccines among IBD patients in our hospital and other hospitals. 

The data from this study will be used to provide specific recommendations to physicians at our center in order to improve vaccination rates. Providers should have conversations with patients to address concerns about influenza and pneumococcal vaccines and give continued education about their role in preventing VPDs. Additional training to nurses and medical assistants regarding educating IBD patients about influenza and pneumococcal vaccines may also be of benefit. Another recommendation is to incorporate continual EMR reminders for the entire medical team caring for IBD patients. This will create supplementary safety nets to ensure providers and patients are receiving this information in a timely manner. An additional recommendation is to confirm that gastroenterologists are taking a full history, including a thorough vaccination history which is being consistently reconciled and updated. 

Strengths of our study include our study population mainly consisting of African American IBD patients, a population often understudied within IBD research. Also, our study location was a safety-net hospital, consisting of mainly low-to-no income patients, again highlighting a patient population not always studied at large academic institutions. Limitations of our study include this is a single-center study with small sample size and our data may not be fully generalizable to all IBD patients. Similar studies should be completed at other safety net institutions with a similar patient population to lessen the likelihood of bias. Future studies will evaluate reasoning for poor vaccine adherence among IBD patients at our center and hospitalization rates for preventable infections due to substandard vaccination history. Additionally, we will compare with current vaccination rates to look for any changes in vaccination uptake since the beginning of the Covid-19 pandemic and review Covid-19 vaccination rates among IBD patients at our center.

## Conclusions

In summary, influenza and pneumococcal vaccination rates of IBD patients at our center were shown to be substandard. Patients were not being consistently vaccinated with PCV13 and/or PPSV23 in accordance with CDC guidelines based on qualifying comorbid medical conditions, in addition to their IBD status, substantially increasing their risk for avoidable hospitalizations and morbidity. Age was also found to play a role in whether patients received the influenza vaccine even though the CDC recommends all patients more than six months of age receive an annual influenza vaccine. While our study was based on current CDC vaccination recommendations, we urge the CDC's Advisory Committee on Immunization Practices (ACIP) to create recommendations more specific to IBD patients, taking into consideration individual factors such as interval and progression of disease and medication regimens, particularly for patients receiving immunosuppressive therapy.
